# Driver Fusions and Their Implications in the Development and Treatment of Human Cancers

**DOI:** 10.1016/j.celrep.2018.03.050

**Published:** 2018-04-03

**Authors:** Qingsong Gao, Wen-Wei Liang, Steven M. Foltz, Gnanavel Mutharasu, Reyka G. Jayasinghe, Song Cao, Wen-Wei Liao, Sheila M. Reynolds, Matthew A. Wyczalkowski, Lijun Yao, Lihua Yu, Sam Q. Sun, The Fusion Analysis Working Group, The Cancer Genome Atlas Research Network, Ken Chen, Alexander J. Lazar, Ryan C. Fields, Michael C. Wendl, Brian A. Van Tine, Ravi Vij, Feng Chen, Matti Nykter, Ilya Shmulevich, Li Ding

**Affiliations:** 1 Department of Medicine, Washington University in St. Louis, St. Louis, MO 63110, USA; 2 McDonnell Genome Institute, Washington University in St. Louis, St. Louis, MO 63108, USA; 3 Institute of Signal Processing, Tampere University of Technology, 33101, Tampere, Finland; 4 Institute for Systems Biology, Seattle, WA 98109, USA; 5 H3 Biomedicine, Inc., Cambridge, MA 02139, USA; 6 Department of Bioinformatics and Computational Biology, The University of Texas MD Anderson Cancer Center, Houston, TX 77230, USA; 7 Departments of Pathology, Genomic Medicine, and Translational Molecular Pathology, The University of Texas MD Anderson Cancer Center, Houston, TX 77230, USA; 8 Department of Surgery, Washington University in St. Louis, St. Louis, MO 63110, USA; 9 Department of Genetics, Washington University in St. Louis, St. Louis, MO 63110, USA; 10 Department of Mathematics, Washington University in St. Louis, St. Louis, MO 63130, USA; 11 Siteman Cancer Center, Washington University in St. Louis, St. Louis, MO 63110, USA; 12 Institute for Biosciences and Medical Technology, University of Tampere, 33520 Tampere, Finland

## Abstract

Gene fusions represent an important class of somatic alterations in cancer. We systematically investigated fusions in 9,624 tumors across 33 cancer types using multiple fusion calling tools. We identified a total of 25,664 fusions, with a 63% validation rate. Integration of gene expression, copy number, and fusion annotation data revealed that fusions involving oncogenes tend to exhibit increased expression, whereas fusions involving tumor suppressors have the opposite effect. For fusions involving kinases, we found 1,275 with an intact kinase domain, the proportion of which varied significantly across cancer types. Our study suggests that fusions drive the development of 16.5% of cancer cases and function as the sole driver in more than 1% of them. Finally, we identified druggable fusions involving genes such as *TMPRSS2*, *RET*, *FGFR3*, *ALK*, and *ESR1* in 6.0% of cases, and we predicted immunogenic peptides, suggesting that fusions may provide leads for targeted drug and immune therapy.

## In Brief

Gao et al. analyze a 9,624 sample TCGA cohort with 33 cancer types to detect gene fusion events. They provide a landscape of fusion events detected, relate fusions to gene expression, focus on kinase fusion structures, examine mutually exclusive mutation and fusion patterns, and highlight fusion druggability.

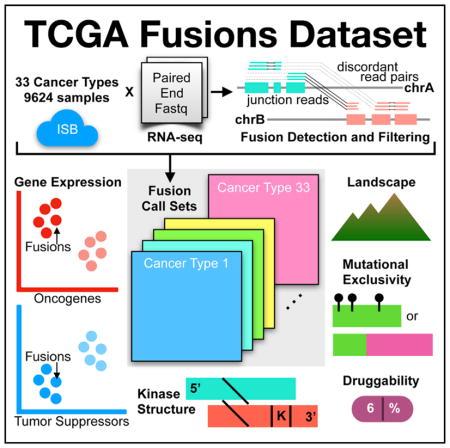

## INTRODUCTION

The ability to determine the full genomic portrait of a patient is a vital prerequisite for making personalized medicine a reality. To date, many studies have focused on determining the landscape of SNPs, insertions, deletions, and copy number alterations in cancer genomes (Kanchi et al., 2014; Kandoth et al., 2013; Kumar-Sinha et al., 2015; Lawrence et al., 2014; Vogelstein et al., 2013; Wang et al., 2014). Although such genomic alterations make up a large fraction of the typical tumor mutation burden, gene fusions also play a critical role in oncogenesis. Gene fusions or translocations have the potential to create chimeric proteins with altered function. These events may also rearrange gene promoters to amplify oncogenic function through protein overexpression or to decrease the expression of tumor suppressor genes.

Gene fusions function as diagnostic markers for specific cancer types. For example, a frequent translocation between chromosomes 11 and 22 creates a fusion between *EWSR1* and *FLI1* in Ewing’s sarcoma. Also, the Philadelphia chromosome 9–22 translocation is characteristic of chronic myeloid leukemia, resulting in the fusion protein *BCR*–*ABL1*. This fusion leads to constitutive protein tyrosine kinase activity and downstream signaling of the PI3K and MAPK pathways, which enables cells to evade apoptosis and achieve increased cell proliferation (Cilloni and Saglio, 2012; Hantschel, 2012; Ren, 2005; Sinclair et al., 2013). Fibrolamellar carcinoma (FLC) in the liver is characterized by a *DNAJB1*–*PRKACA* fusion. A recent study of The Cancer Genome Atlas (TCGA) tumors revealed this fusion transcript is specific to FLC, differentiating it from other liver cancer samples (Dinh et al., 2017). In contrast, *FGFR3*–*TACC3* is an inframe activating kinase fusion found in multiple cancer types, including glioblastoma multiforme (GBM) (Lasorella et al., 2017; Singh et al., 2012) and urothelial bladder carcinomas (BLCA) (Cancer Genome Atlas Research Network, 2014). Other recurrent fusions have also been reported in multiple cancer types (Bass et al., 2011; Jones et al., 2008; Palanisamy et al., 2010), and functional characterization of a few selected fusion genes in cellular model systems has confirmed their oncogenic nature (Lu et al., 2017).

Recently, large-scale genomic studies have used the TCGA RNA sequencing (RNA-seq) data corpus to systematically identify and compile fusion candidates across many cancer types. For example, as part of its goal to develop a comprehensive, genome-wide database of fusion genes, ChimerDB (Lee et al., 2017) has analyzed RNA-seq data of several thousand TCGA cases. Giacomini et al. (2013) performed breakpoint analysis on exon microarrays across 974 cancer samples and identified 198 candidate fusions in annotated cancer genes. A searchable portal of TCGA data includes 20,731 fusions called from 9,966 cancer and 648 normal samples (Hu et al., 2018). Some studies focus on important classes of genes, such as kinase fusions (Stransky et al., 2014), which may have particular structural properties that are selected for during oncogenesis and cancer progression. However, most efforts have used only a single fusion calling algorithm. Because disagreements among different callers are common, there is a need to develop a comprehensive approach that combines the strengths of various callers to achieve higher fusion calling accuracy. Furthermore, large-scale analyses are likely to expand the targetable landscape of fusions in cancer, revealing potential treatment options for patients.

Here, we leverage multiple newly developed bioinformatic tools to methodically identify fusion transcripts across the TCGA RNA-seq data corpus using the Institute for Systems Biology (ISB) Cancer Genomics Cloud. These tools include STAR-Fusion, Breakfast, and EricScript (STAR Methods). Fusion calling across 9,624 TCGA tumor samples from 33 cancer types identified a total of 25,664 fusion transcripts, with a 63.3% validation rate for the samples having available whole-genome sequencing data. Furthermore, we investigated the relationship between fusion status and gene expression, the spectrum of kinase fusions, mutations, and fusions found in driver genes, and fusions as potential drug and immunotherapy targets.

## RESULTS

### Fusion Detection Pipeline and WGS-Based Validation of a Subset of Fusion Predictions

**Figure 1:**
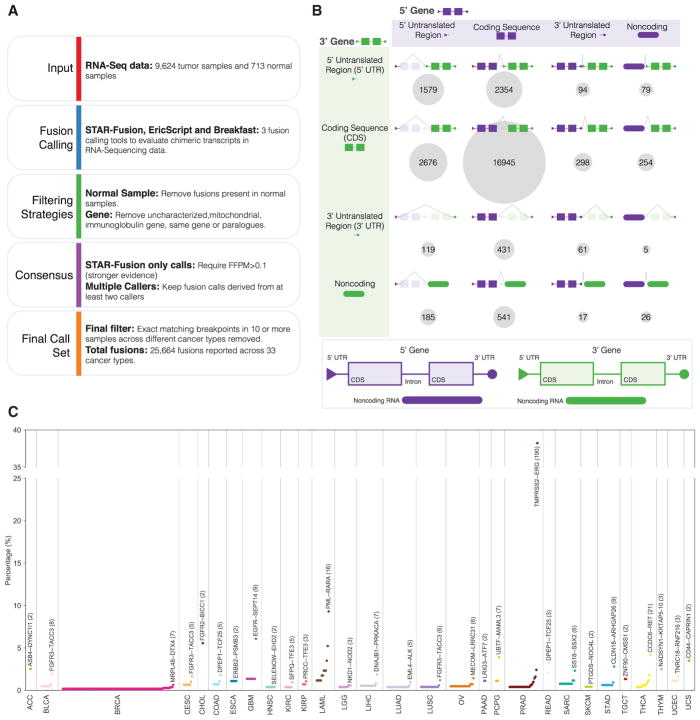
Fusion Detection and Landscape in Cancer (A) Fusion calling and filtering pipeline. (B) Cartoon overview of fusion gene partner breakpoints. Purple indicates the 5′ gene partner, and green indicates the 3′ gene partner. For both the 5′ and 3′ gene partners, fusion gene breakpoints can occur in the following genomic regions: 5′ UTR (triangle), coding sequence (CDS; rectangle), 3′ UTR (circle), and non-coding region (rounded rectangle). For each fusion event, a dotted line connects the breakpoints in the 5′ and 3′ gene partners to create the predicted fusion and the circle size, while number represents the total fusion events classified into the associated fusion category. (C) The dot plot shows the frequency of recurrent fusions found in each cancer type. The most recurrent fusion in each cancer type is labeled. Cancer types without recurrent fusions are not shown.

We analyzed RNA-seq data from 9,624 tumor samples and 713 normal samples from TCGA using STAR-Fusion (STAR Methods), EricScript (Benelli et al., 2012), and Breakfast (STAR Methods; Table S1). A total of 25,664 fusions were identified after extensive filtering using several panel-of-normals databases, including fusions reported in TCGA normal samples, GTEx tissues (Consortium, 2013) and non-cancer cells (Babiceanu et al., 2016) (STAR Methods; Figure 1A; Table S1). Our pipeline detected 405 of 424 events curated from individual TCGA marker papers (Table S1) (95.5% sensitivity).

We further cross-confirmed our transcriptome sequencing-based fusion detection pipeline by incorporating whole-genome sequencing (WGS) data, where available. WGS paired-end reads aligned to the partner genes of each fusion were used to validate fusions detected using RNA-seq. Using all available WGS, including both low-pass and high-pass data, from 1,725 of the 9,624 cancer samples across 25 cancer types, we were able to evaluate 18.2% (4,675 fusions) of our entire fusion call set. Of that subset, WGS validated 63.3% of RNA-seq-based fusions by requiring at least three supporting discordant read pairs from the WGS data (Figure S1).

### Fusion Landscape across 33 Cancer Types

Categorizing the 25,664 fusions on the basis of their breakpoints, we found that the majority of breakpoints are in coding regions (CDS) of both partner genes (Figure 1B). Surprisingly, there are many more fusions in 5′ UTRs compared with 3′ UTRs for both partner genes, given that 3′ UTRs are generally longer (Mann-Whitney U test, p < 2.2e-16). This could be explained by having more open chromatin in the 5′ UTR region (Boyle et al., 2008), the larger number of exons in 5′ UTRs than 3′UTRs (Mann-Whitney U test, p < 2.2e-16) (Mignone et al., 2002), but could also indicate some regulatory mechanisms, such as alternative use of the promoter region of a partner gene.

For different cancer types, the total number of fusions per sample varies from 0 to 60, with a median value of 1 (Figure S1). Cancer types with the fewest number of fusions per sample are kidney chromophobe (KICH), kidney renal clear cell carcinoma (KIRC), kidney renal papillary cell carcinoma (KIRP), low-grade glioma (LGG), pheochromocytoma and paraganglioma (PCPG), testicular germ cell tumors (TGCT), thyroid carcinoma (THCA), thymoma (THYM), and uveal melanoma (UVM), each with a median of 0. Other cancer types show a range of medians between 0.5 and 5 fusions per sample, although most samples demonstrate zero or only one inframe, disruptive fusion relevant to oncogenesis.

Frequencies of recurrent fusions found in each cancer are illustrated in Figure 1C (Table S1). The most recurrent example within any cancer type was *TMPRSS2*–*ERG* in prostate adenocarcinoma (PRAD; 38.2%). We found *FGFR3*–*TACC3* to be the most recurrent fusion in BLCA (2.0%), cervical squamous cell carcinoma and endocervical adenocarcinoma (CESC, 1.7%), and lung squamous cell carcinoma (LUSC, 1.2%). Other top recurrent fusions include *EML4*–*ALK* in lung adenocarcinoma (LUAD; 1.0%), *CCDC6*–*RET* in THCA (4.2%), and *FGFR2*–*BICC1* in cholangiocarcinoma (CHOL; 5.6%).

### Fusion Gene Expression in Oncogenes and Tumor Suppressors

Fusion events may be associated with altered expression of one or both of the fusion gene partners, a well-known example being multiple myeloma tumors in which translocation t(4;14) fuses the highly expressed IGH locus with the tyrosine protein kinase *FGFR3* (Manier et al., 2017). We integrated gene expression, copy number, and fusion annotations to systematically test for associations between gene expression and fusion status.

**Figure 2:**
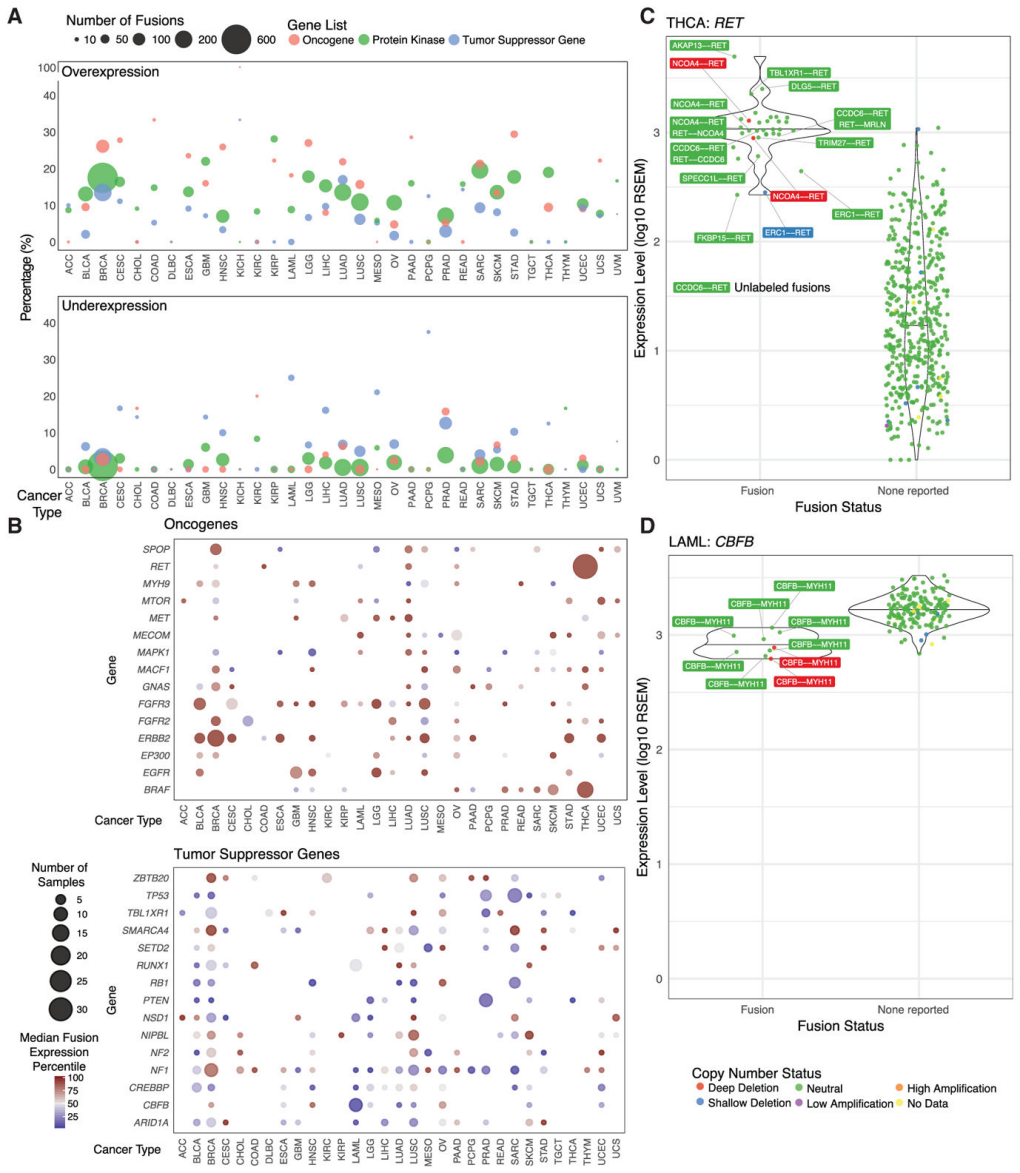
Fusion Expression Outliers (A) The dot plot indicates the percentage of fusions called in which one of the partner genes is an expression outlier (overexpression or underexpression). The size of the dot corresponds to the number of fusions called in each cancer type. Color corresponds to genes of interest coming from lists of oncogenes, protein kinases, and tumor suppressor genes. (B) The dot plot shows the relative expression level of samples with fusions compared with those without fusions. Each sample has a particular expression percentile at a given gene, and color indicates the median percentile of samples with a fusion in that gene. Genes are the 15 most recurrent oncogenes and tumor suppressor genes. Size corresponds to the number of samples in each cancer type with a fusion at that gene. (C and D) Expression of samples at *RET* and *CBFB* in thyroid carcinoma (THCA) (C) and acute myeloid leukemia (LAML) (D), respectively. Color indicates a categorical copy number ranging from deep deletion to high amplification.

For each fusion having an oncogene, kinase, or tumor suppressor gene (TSG) (Table S2), we determined whether that sample was an expression outlier for that gene and subsequently examined resulting percentages of both under- and overexpressed genes in each cancer type (Table S3). Figure 2A shows that between 6% (mesothelioma [MESO]) and 28% (KIRP) of kinase fusions displayed outlier overexpression of the kinase partner. Oncogenes tended to show higher likelihoods of overexpression, whereas TSGs displayed lower likelihoods. Between 3% (breast invasive carcinoma [BRCA]) and 38% (PCPG) of TSG fusions showed outlier underexpression, generally higher than both oncogenes and kinases.

Figure 2B illustrates the median percentile expression levels of the most highly recurrent oncogenes and TSGs involved in fusions (Table S3). Samples with fusions involving oncogenes, such as *EGFR*, *ERBB2*, and *RET*, showed increased expression of those genes relative to samples without fusions across cancer types. Most TSGs showed inconsistent patterns of expression across cancer types. However, the global trend for TSGs is decreased expression compared with non-fusion samples.

We also examined the relationship between TSG mutations and fusions to determine whether frequently fused TSGs were also disrupted by other mutation types. A variety of patterns were noted. For example, *TP53* is affected by mutations rather than fusions in most cancer types. However, in sarcoma (SARC), both fusions and mutations affecting TP53 were detected. In acute myeloid leukemia (LAML), several *CBFB* fusions but no mutations were observed, yet other cancer types also exhibited *CBFB* mutations (Table S3; Figure S2). Our results suggest that alternative mechanisms are used by tumor cells in a cancer type-specific manner.

We also observed associations between fusion status and expression level in well-known fusions (Table S3), such as *RET*–*NTRK1* in thyroid cancer, *EML4*–*ALK* in lung cancer (Stransky et al., 2014), and *DNAJB1*–*PRKACA* in the FLC subtype of liver cancer (Dinh et al., 2017). *RET* fusions in thyroid THCA and LUAD are inframe protein kinase fusions with overexpression of the 3′ *RET* oncogene (Figure 2C). Recurrent *CBFB*–*MYH11* fusions in LAML are significantly associated with decreased expression of the tumor suppressor *CBFB*, which functions as a transcriptional regulator (Haferlach et al., 2010) (Figure 2D).

In breast cancer, copy number amplification is a well-known mechanism of *ERBB2* overexpression, and treatment of these HER2^+^ patients with trastuzumab is an established and effective targeted therapy (Smith et al., 2007). Interestingly, three of four samples with *ERBB2* fusions and two samples without a called fusion showed HPV integration within 1 Mb of *ERBB2* (Cao et al., 2016). *ERBB2* fusion gene partners *PPP1R1B* and *IKZF3* are genomic neighbors of *ERBB2*, suggesting that these fusions could be a by-product of local instability, potentially induced by the viral integration and subsequent breakage fusion events. By careful analysis of the association between fusions and expression, we have identified strategies for improving both sensitivity and specificity of fusion calls.

### Structure and Spectrum of Kinase Fusions

**Figure 3:**
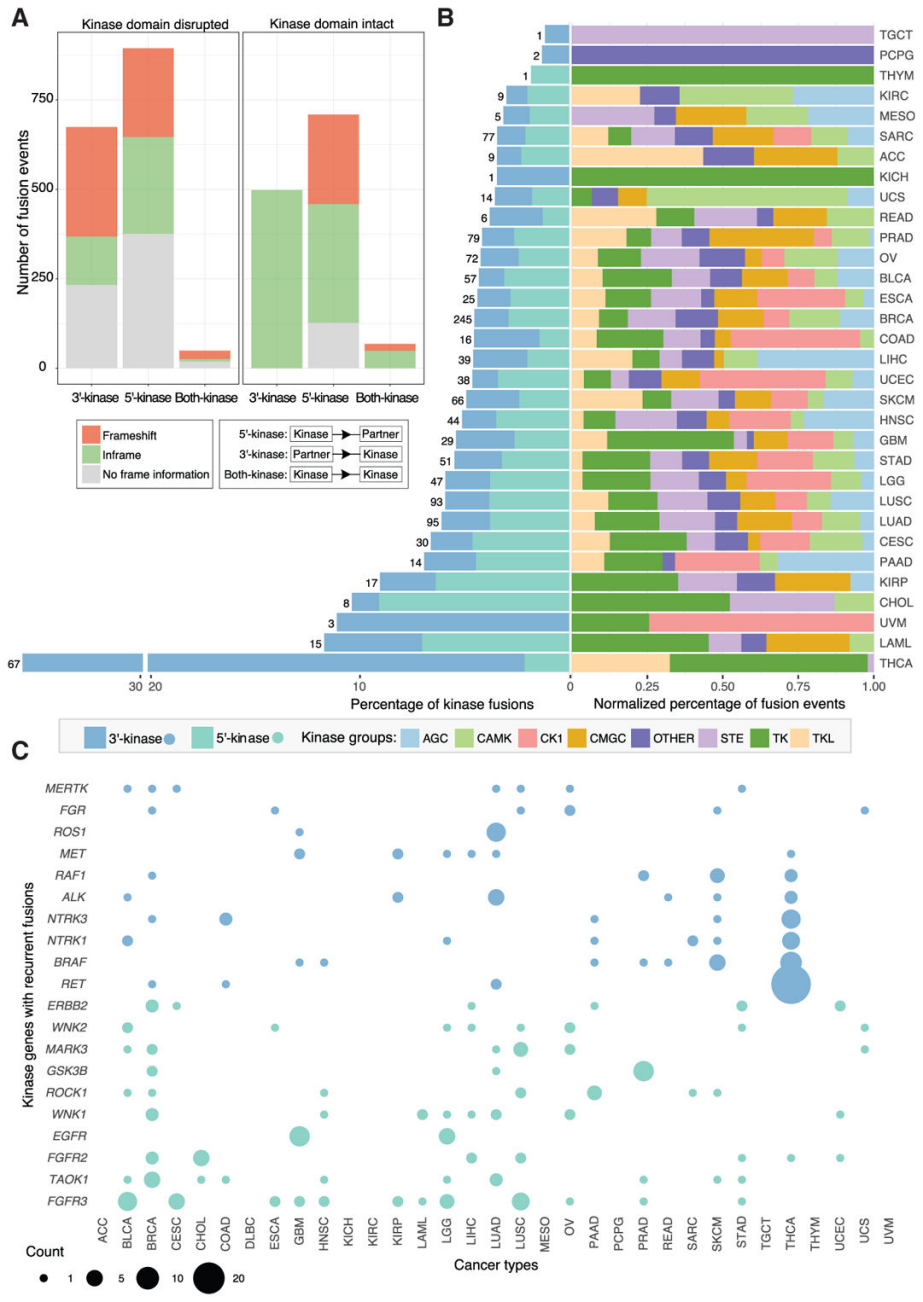
Protein Kinase Fusions (A) The bar chart indicates the number of protein kinase fusions with the kinase at the 5′ or 3′ end, inframe or frameshift, and kinase domain intact or disrupted. (B) The left bar plot shows the percentage of samples with kinase fusions across different cancer types. The number of samples with a kinase fusion is also indicated at the end of each bar. Light green and blue denote 5′ kinase and 3′ kinase fusions, respectively. The right bar plot shows the normalized percentage of kinase fusions broken down by kinase groups. (C) The dot plot shows the numbers of samples for recurrent fusions across different cancer types. Light green and blue denote 5′ kinase and 3′ kinase fusions, respectively.

Some oncogenic kinase fusions are susceptible to kinase inhibitors (Stransky et al., 2014), suggesting that additional therapeutic candidates might be discovered by examining fusion transcripts involving protein kinase genes. In total, we detected 2,892 such events, comprising 1,172 with kinase at the 3′ end (3′-kinase), 1,603 with kinase at the 5′ end (5′-kinase), and 117 with both partners being kinases (both-kinase) (Figure 3A; Table S4). Analysis of the catalytic kinase domains using the UniProt/PFAM domain database (STAR Methods) showed that 1,275 kinase fusions (44.1%) retained an intact kinase domain (Figure 3A). We further predicted open reading frames for these fusions and separated them into three categories with respect to the frame of the 3′ gene: inframe, frameshift, and no frame information (e.g., breakpoint at UTR, intron, or non-coding RNA). In general, there were more inframe fusions than frameshift fusions, especially for 3′-kinase fusions, because preserving the reading frame is required to keep the kinase domain intact. For subsequent kinase analyses, we focused only on those 1,275 fusions with intact domains, further classifying the both-kinase group into 3′-kinase or 5′-kinase on the basis of the position of the intact domain.

Comparison of kinase fusions across different cancer types indicated that kinase fusions are significantly enriched in THCA (35.6%, Fisher’s exact test, p < 2.2e-16) (Figure 3B). Moreover, the majority were 3′-kinase fusions (94.0%), a significantly higher percentage than what we observed in other cancer types (Fisher’s exact test, p < 2.2e-16). We further divided these fusions into eight categories on the basis of different kinase groups, including AGC, CAMK, CK1, CMGC, STE, TK, and TKL. In general, we found that the percentages of different categories vary across cancer types (Figure 3B). For example, there are more TK fusions in THCA and GBM, more CK1 fusions in uterine corpus endometrial carcinoma (UCEC), colon adenocarcinoma (COAD), and esophageal carcinoma (ESCA) and more AGC fusions in liver hepatocellular carcinoma (LIHC). Across different cancer types, we found an enrichment of TK and TKL kinase fusions for 3′-kinases but no strong preference for 5′-kinases (Figure S3).

Recurrent kinase fusions are of great interest as potential drug targets. Overall, we detected 744 5′-kinase and 531 3′-kinase fusions. Of these, 147 and 99 were recurrent, respectively, mostly across cancer types rather than within cancer types (Figure S3). As expected, fusions in the *FGFR* kinase family (*FGFR2* and *FGFR3*) are the most frequent 5′-kinase fusions, given their high recurrence in individual cancer types (Figure 3C). *WNK* kinase family fusions (*WNK1* and *WNK2*) were also detected in multiple cancer types. The *WNK* family is phylogenetically distinct from the major kinase families, and there is emerging evidence of its role in cancer development (Moniz and Jordan, 2010). Here, we found a total of 23 WNK-family fusions, most of which resulted in higher expression of *WNK* mRNA (Figure S4). The increased expression was not generally accompanied by copy number amplification; for example, neither *WNK1* nor *WNK2* was amplified in ESCA or LIHC. Incidentally, *ERC1–WNK1* was also detected recently in an independent Chinese esophageal cancer cohort (Chang et al., 2017). For 3′-kinase fusions, all the top ten kinase genes are tyrosine kinases, most of which are enriched in THCA, including *RET*, *BRAF*, *NTRK1*, *NTRK3*, *ALK,* and *REF1* (Figure 3C). *FGR* fusions were found in seven samples the same partner gene *WASF2*, five of which showed higher expression of *FGR* gene. In these five samples, the breakpoints for the two genes are the same (5′UTR of both genes) resulting in usage of the stronger *WASF2* promoter for the *FGR* gene. Interestingly, recurrent *MERTK* fusions are singletons in each individual cancer type with *TMEM87B*, and *PRKACA* fusions are observed only in liver cancer with *DNAJB1* (Figure S3).

**Figure 4:**
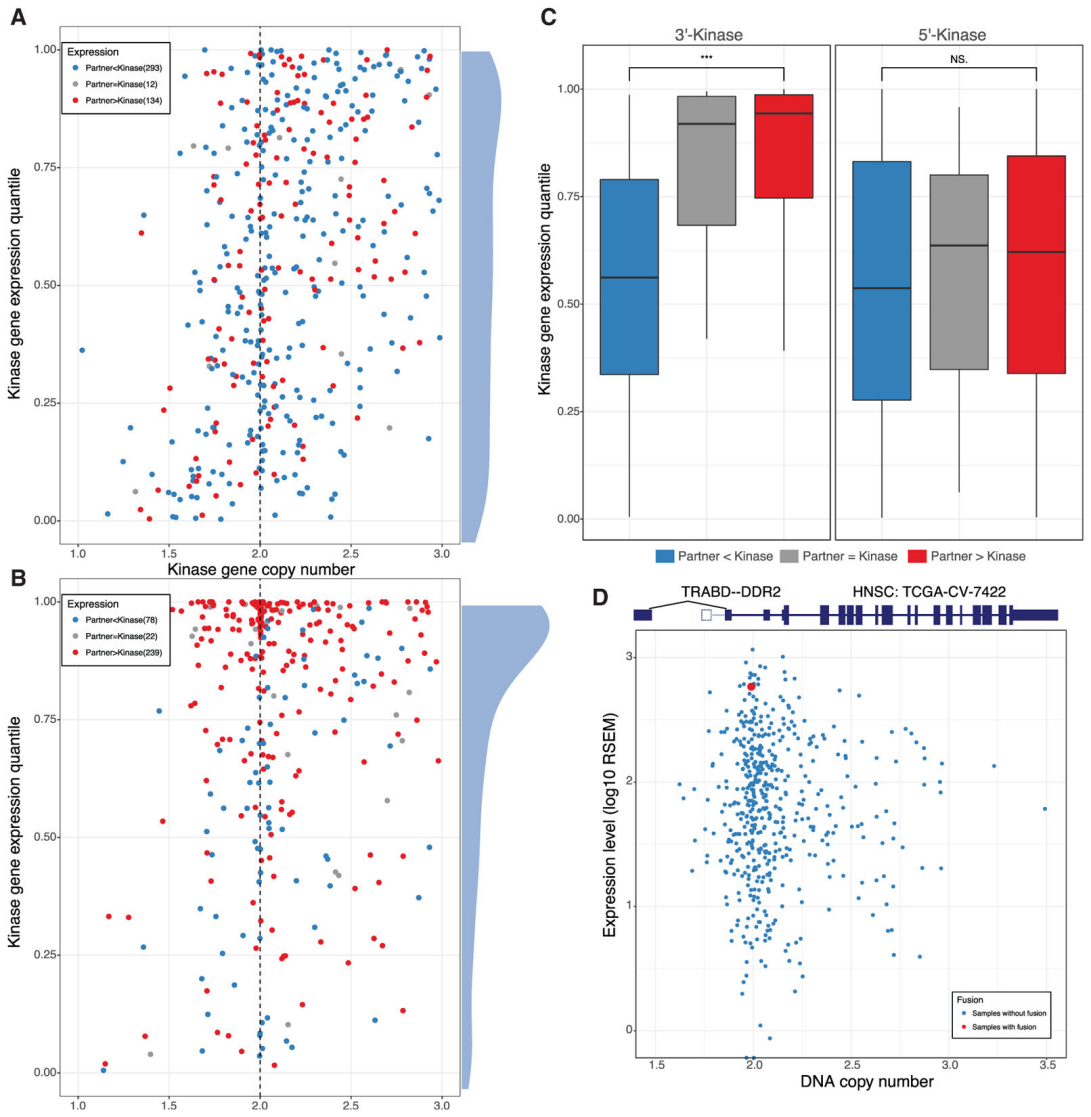
Kinase Gene Expression Regulated by Fusion (A) The scatterplot shows the gene expression quantile (y axis) for the 5′-kinase without copy number variation (between one and three copies; x axis). All genes are classified among three categories: kinase expression higher, equal, and lower, compared with partner expression, marked in blue, gray, and red, respectively. The density plot for expression quantile is also shown on the right. (B) The scatterplot shows the gene expression quantile (y axis) for the 3′-kinase without copy number variation (between one and three copies; x axis). The colors represent the same three categories as (A). The density plot for expression quantile is also shown. (C) Boxplot comparing the distribution of kinase gene expression quantile between the three groups defined in (A) for 5′-kinase and 3′-kinase, respectively. (D) Schematic of *TBABD–DDR2* fusion gene structure in an HNSC sample and scatterplot of *DDR2* copy number versus mRNA expression in HNSC. The samples with and without this fusion are marked in red and blue, respectively.

To further understand the regulation of kinase fusions, we compared the gene expression patterns between the kinase gene and partner gene. There are in total 1,035 kinase fusions with both gene expression and copy number data available. To control for the effect of copy number amplification on gene expression, we focused on the fusions with copy numbers between 1 and 3, including 439 5′-kinase and 339 3′-kinase fusions (Figures 4A and 4B). For 5′-kinase fusions, the kinase gene expression quantiles are uniformly distributed, indicating that the kinase gene expressions in the samples with fusion are not significantly different from the samples without fusion (Figure 4A). However, 3′-kinase genes tend to show higher expression in samples with a fusion compared with the ones without. To explain this, we classified the fusion events into three categories on the basis of the relative expression pattern between the kinase gene and its partner in samples from the same cancer type. Most (66.7% [293 of 439]) 5′-kinase fusions showed lower expression in the partner gene compared with the kinase. In contrast, 70.5% of 3′-kinase fusions (239 of 339) showed higher partner expression (Figures 4A and 4B). Moreover, those 3′-kinase fusions involving a more highly expressed 5′ partner also show higher kinase expression (Figure 4C). For example, we found a *TRABD–DDR2* fusion in one head and neck squamous cell carcinoma (HNSC) sample, which fused the stronger *TRABD* promoter with *DDR2*, resulting in its overexpression (Figure 4D). This patient could potentially be treated using dasatinib, which targets overexpressed *DDR2* in HNSC (von Massenhausen et al., 2016). *DDR2* fusions were also detected in another nine samples from five different cancer types, which could be treated similarly given sufficient DDR2 overexpression (Table S1).

### Mutual Exclusivity between Fusions and Mutations

Although mutations in oncogenes or TSGs may lead to tumorigenesis, fusions involving those genes are also an important class of cancer driver events. We systematically profiled mutations and fusions in 299 cancer driver genes (Table S2; Bailey et al., 2018) to assess the contributions of fusion genes in carcinogenesis in the 8,955 TCGA patients who overlap between the mutation call set (Key Resources Table, Public MC3 MAF; Ellrott et al., 2018) and our fusion call set. We characterized patients as having a driver mutation, a mutation in a driver gene, and/or a driver fusion (fusion involving a driver gene).

**Figure 5:**
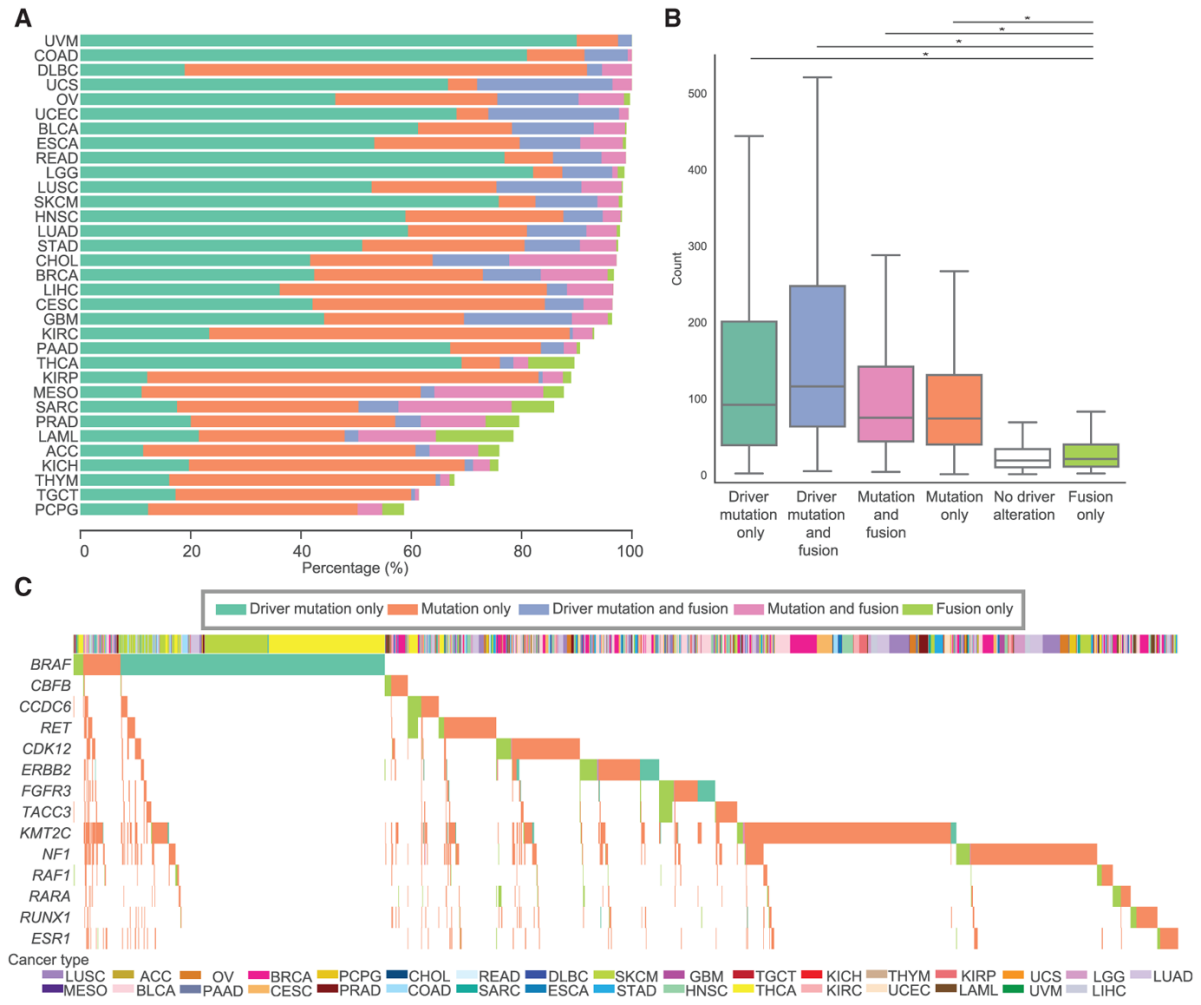
Mutual Exclusivity between Driver Mutations and Driver Fusions (A) The bar plot shows the percentages of samples with driver mutations only (green), mutations only (orange), driver mutation and fusion (blue), mutation and fusion (pink), or fusion only (light green) events in 299 cancer driver genes. (B) Distribution of mutation burden across each alteration group designated in all figures. (C) All samples with fusions or mutations in any of the genes indicated on the left are displayed on the x axis. For each gene, samples are clustered by the alteration group. Bottom bar indicates cancer type.

Although the majority of cancer cases have known driver mutations (48.6%, mean 6.8 mutations) or mutations in driver genes (28.1%, mean 4.2 mutations), we found that 8.3% have both driver mutations and driver fusion events (mean 5.5 mutations and 1.2 fusions), 6.4% have both mutations and fusions in driver genes (mean 4.2 mutations and 1.3 fusions), and 1.8% have driver fusions only (mean 1.1 fusions) (Figure 5A). This distribution is consistent with the notion that only a few driver events are required for tumor development (Kandoth et al., 2013).

We further examined the total number of mutations for samples and observed a low mutational burden in the group with driver fusion only, which is comparable with the group with no driver alterations (Figure 5B). The significant decrease in the numbers of mutations (Mann-Whitney U test, p < 2.2e-16) reflects the functionality of fusions across multiple cancer types. Moreover, within cancer types, we observed a range of 0.2% (HNSC) to 14.0% (LAML) of tumors with fusions but no driver gene mutations. Among those LAML tumors that have fusions and no driver gene mutations, we identified several well-recognized fusions relevant to leukemia, such as *CBFB*–*MYH11* (number of samples = 3), *BCR*–*ABL1* (n = 2), and *PML*–*RAR* (n = 2). We also identified the leukemia-initiating fusion *NUP98*–*NSD1* in two LAML tumors (Cancer Genome Atlas Research Network et al., 2013b).

We then examined the relationship of fusions and mutations in the same driver gene (Figure 5C). The result shows that when fusion events are present in a gene, mutations in the same gene are rarely found, supporting a pattern of mutual exclusivity of the two types of genomic alteration. This trend was observed across many patients and many cancer types. Our results suggest that a considerable number of tumors are driven primarily or solely by fusion events.

### Contributions of Fusions to Cancer Treatment

**Figure 6:**
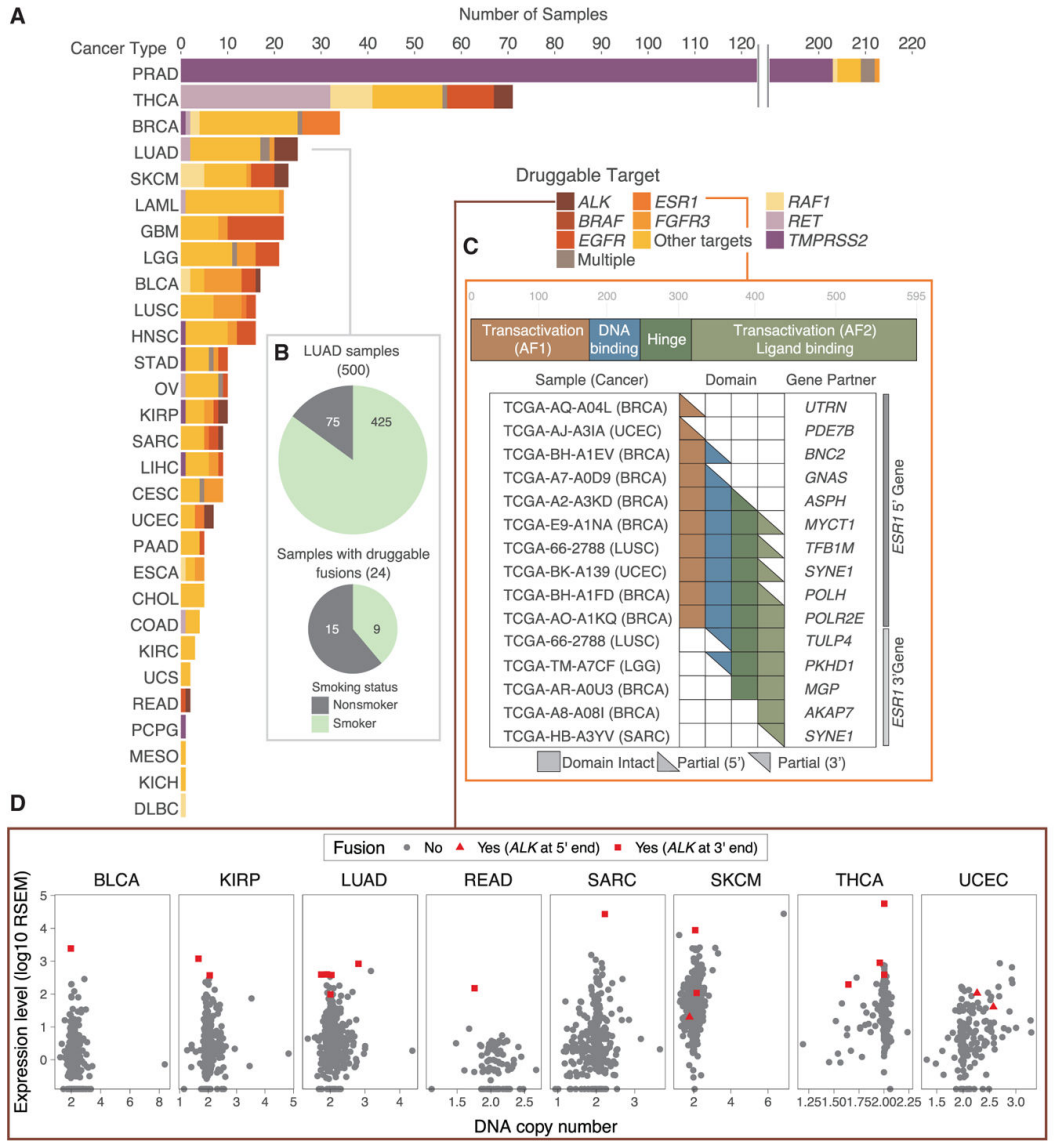
Druggable Fusion Targets (A) The bar chart indicates the number of samples potentially treatable on the basis of their fusion status. (B) Percentages of LUAD samples with known smoking status. (C) *ESR1* domains kept in *ESR1* fusions across cancer types. (D) *ALK* expression across cancer types indicating *ALK* fusion status.

We investigated potentially druggable fusion events in our call set using our curated Database of Evidence for Precision Oncology (DEPO; Sun et al., unpublished data) (Table S5). We defined a fusion as druggable if there is literature supporting the use of a drug against that fusion, regardless of cancer type (allowing for “off-label” drug treatment). We found potentially druggable fusions across 29 cancer types, with major recurrent druggable targets in PRAD (*TMPRSS2*, 205 samples), THCA (*RET*, 33 samples), and LAML (*PML*–*RARA*, 16 samples) (Figure 6A). *FGFR3* was a potential target (both on-label and off-label) in 15 cancer types. Overall, we found 6.0% of samples (574 of 9,624 samples) to be potentially druggable by one or more fusion targeted treatments. Further study of fusions in human cancer will facilitate the development of precision cancer treatments.

We analyzed patterns of fusion druggability in LUAD, stratifying by smoking status. In this dataset, 15% of LUAD samples (75 of 500 samples with known smoking status) were from never smokers, while a significantly higher percentage of never smokers (15 of 75 samples) versus smokers (9 of 425 samples) were found to have druggable fusion (chi-square test, p < 1e-6) (Figure 6B). Several Food and Drug Administration (FDA)-approved drugs exist to target *ALK* fusions in lung and other cancer types. We observed *ALK* fusions in 20 samples from eight cancer types (5 samples in LUAD). In most cases, fusion status corresponded to copy number neutral overexpression of *ALK* (Figure 6D). In 17 of 20 cases, *ALK* was the 3′ partner of the fusion pair, with *EML4* being the most frequent 5′ partner (7 of 17).

*ESR1* encodes an estrogen receptor with important and drug-gable relevance to breast cancer (Li et al., 2013). We detected *ESR1* fusions in 16 samples from five different cancer types (9 samples from BRCA). Of the 9 BRCA samples, 8 are known be from the luminal A or B subtype. We observed strict mutual exclusivity between *ESR1* mutations and fusions (Figure 5C). Of the 16 fusions, 11 have *ESR1* at the 5′ end and 5 at the 3′ end. When *ESR1* is the 5′ gene in the fusion, the transactivation (AF1) domain is always included (Figure 6D). When *ESR1* is the 3′ gene, the transactivation (AF2) domain is always included. Those samples with *ESR1* fusion tend of have higher *ESR1* expression, especially in the 9 BRCA samples (Figure S5). Similarly, *ESR1* expression is higher when *ESR1* is mutated in BRCA, CESC, and UCEC, which are all hormone receptor-related cancer types (Cancer Genome Atlas, 2012; Cancer Genome Atlas Research Network et al., 2013a, 2017). Further functional study to determine the mechanism of *ESR1* fusions could suggest drug development directions.

Immunotherapy based on tumor-specific neoantigens shows promise in treating cancer patients (Bobisse et al., 2016). Gene fusions found in tumor cells can generate peptides, which may serve as neoantigen candidates. However, patients with known driver fusions may be poor candidates for immunotherapy because of their reduced mutational burden, especially without clear evidence of immune cell infiltration and overall immunogenicity. As an exploratory and speculative analysis, we investigated neoantigens produced by gene fusions (Andreatta and Nielsen, 2016). On average, there were 1.5 predicted neoantigens per fusion across different cancer types (Figure S6; Table S5). The mean number of predicted neoantigens per fusion ranged from 0.33 in KICH to 2.88 in THYM. We also compared the number of neoantigens for inframe and frameshift fusions (Figure S6). Results show that frameshift fusions can generate more immunogenic epitopes than inframe fusions (mean value 2.2 versus 1.0), though nonsense-mediated decay might reduce some of this potential difference.

We further investigated seven fusions for which there were at least four samples having one or more neoantigen candidates (Figure S6). In particular, *TMPRSS2*–*ERG*, *CCDC6*–*RET*, and *FGFR3*–*TACC3* have the highest number of samples with predicted neoantigen candidates. Our results show that the fusion product is only immunogenic in a small subset of patients, especially for *TMPRSS2*–*ERG* fusions. Again, without clear evidence of immune cell infiltration and overall immunogenicity, any fusion neoantigen analysis remains exploratory and speculative.

## DISCUSSION

In this study, we applied multiple RNA-seq fusion callers, namely STAR-Fusion, EricScript, and Breakfast, followed by a stringent filtering strategy, to identify potential driver fusion events across 33 cancer types. We were able to successfully identify 95.5% of fusions reported in TCGA marker papers. Although existing studies have published fusion calls across the TCGA cancer cohort (Hu et al., 2018; Stransky et al., 2014), we have improved on prior analyses by integrating results across multiple fusion callers and by applying stringent filtering to derive a confident dataset of fusion events from 9,624 tumor samples. Importantly, we investigated the biology and evaluated the significance of fusions in the cancer context. Of the 25,664 fusions we detected, 18.2% could be tested for validation using available WGS data, leading to a 63.3% validation rate.

By integrating gene expression, copy number, and fusion annotation data, we evaluated the biological and therapeutic implications of fusion events. Kinase- and oncogene-related fusions tended to be overexpression outliers, whereas fusions involving TSGs showed the opposite effect overall. When comparing fusion events with the remainder of the cancer cohort, fusions involving oncogenes such as *EGFR*, *ERBB2*, and *RET* had increased expression. Overexpressed fusions, especially inframe kinase fusions, are commonly targeted for therapy because of their susceptibility to kinase inhibitors.

For all 2,892 kinase fusions, we translated the resulting peptide sequence, finding that 1,275 had functional catalytic kinase domains. Comparison of kinase fusions across different cancer types showed that THCA has significantly more kinase fusions, most of which were 3′ kinase fusions. In addition to well-known recurrent fusions such as *FGFR3–TACC3*, we also detected 245 kinases with recurrent fusions to different partner genes, which may ultimately prove to be successful drug targets.

We showed that a meaningful percentage of patients (1.8%) harbor fusions involving cancer driver genes but have no driver gene mutations. Notably, 6.0% of cancer patients could potentially benefit from existing drugs targeting fusion products. Moreover, our analysis also highlights an important consideration for immunotherapy treatment in patients with fusions. The significant decrease in mutational burden observed in patients with fusions in driver genes points toward a reduced efficacy of immunotherapy in these patients, despite fusion peptides themselves potentially being good immunogenic targets. Many fusions are already known to be drug targets.

Our study demonstrates the necessity of performing fusion analysis across multiple cancer types. Our approach integrated the results of multiple fusion calling algorithms, lending confidence to fusions with lower levels of RNA-seq read support that might otherwise have been discarded. We sought to prioritize fusions relevant to cancer by highlighting their associations with gene expression, potential for targeted therapy, and roles in cancer hallmark pathways. Fusion allele frequency is an elusive measure from RNA-seq data, and tracking the clonal evolution of fusions within a tumor remains an exciting opportunity for study. Fusions play an increasingly appreciated role in tumorigenesis and progression and represent an important source of improved treatment options. Ultimately, our multi-tool, integrative bioinformatic detection approach helps define the universe of fusions in cancer. Furthermore, it reminds us that developing robust and widely applicable clinical diagnostic approaches that can document fusions across cancer types is vital. Such approaches are critical to identifying those patients who can benefit from both established treatments and clinical trials.

## STAR★METHODS

### KEY RESOURCES TABLE

**Table:** 

REAGENT or RESOURCE	SOURCE	IDENTIFIER
Deposited Data		
Public MC3 MAF	Ellrott et al., 2018	https://gdc.cancer.gov/about-data/publications/
UniProt/PFAM domain database	See link	http://www.uniprot.org/database/DB-0073
Database of Precision Oncology	See link	http://dinglab.wustl.edu/depo
Essential Genes/Drivers genes used	Bailey et al., 2018	Table S2; https://gdc.cancer.gov/about-data/publications
ISB Cancer Genomics Cloud	See link	https://isb-cgc.appspot.com/
Software and Algorithms		
STAR-Fusion	Hass et al., 2017	https://github.com/STAR-Fusion/STAR-Fusion/wiki
EricScript	Benelli et al., 2012	https://sites.google.com/site/bioericscript/
Breakfast	See link	https://github.com/annalam/breakfast
AGFusion	Murphy and Elemento, 2016	https://github.com/murphycj/AGFusion

### CONTACT FOR REAGENT AND RESOURCE SHARING

For further information regarding data, please contact Li Ding (lding@wustl.edu).

### EXPERIMENTAL MODEL AND SUBJECT DETAILS

TCGA collected both tumor and non-tumor biospecimens from human samples (https://cancergenome.nih.gov/abouttcga/policies/informedconsent).

### METHOD DETAILS

#### Dataset Description

Aligned RNA-seq bam files were analyzed using the ISB Cancer Genomics Cloud (https://isb-cgc.appspot.com/). These 33 cancer types included in this study are adrenocortical carcinoma [ACC], bladder urothelial carcinoma [BLCA], brain lower grade glioma [LGG], breast invasive carcinoma [BRCA], cervical squamous cell carcinoma and endocervical adenocarcinoma [CESC], cholangiocarcinoma [CHOL], colon adenocarcinoma [COAD], esophageal carcinoma [ESCA], glioblastoma multiforme [GBM], head and neck squamous cell carcinoma [HNSC], kidney chromophobe [KICH], kidney renal clear cell carcinoma [KIRC], kidney renal papillary cell carcinoma [KIRP], acute myeloid leukemia [LAML], liver hepatocellular carcinoma [LIHC], lung adenocarcinoma [LUAD], lung squamous cell carcinoma [LUSC], lymphoid neoplasm diffuse large B cell lymphoma [DLBC], mesothelioma [MESO], ovarian serous cystadenocarcinoma [OV], pancreatic adenocarcinoma [PAAD], pheochromocytoma and paraganglioma [PCPG], prostate adenocarcinoma [PRAD], rectum adenocarcinoma [READ], sarcoma [SARC], skin cutaneous melanoma [SKCM], stomach adenocarcinoma [STAD], testicular germ cell tumors [TGCT], thymoma [THYM], thyroid carcinoma [THCA], uterine carcinosarcoma [UCS], uterine corpus endometrial carcinoma [UCEC], and uveal melanoma [UVM]. The sample set consists of 10,337 total TCGA samples, 9,624 tumor samples, and 713 normal samples.

Level-3 gene expression (RSEM) and segment-based copy number data were downloaded from Broad GDAC firehose (https://gdac.broadinstitute.org) (version: 2016_01_28). Gene-based copy number data were obtained by intersecting with RefSeq gene annotation bed file (version: 2013-07-27). Mutation calls were provided by the Multi-Center Mutation Calling in Multiple Cancers (MC3) working group within TCGA (Key Resources Table; Ellrott et al., 2018).

#### Fusion Detection and Filtering

TCGA RNA-seq data were downloaded from Cancer Genomics Hub (CGHub, https://cghub.ucsc.edu) and analyzed using the ISB Cancer Genomics Cloud (https://isb-cgc.appspot.com/). For each sample, the fastq file was mapped to the human genome (build 38) followed by fusion calling using STAR-Fusion (parameters:–annotation–coding-effect), EricScript (default parameters) (https://sites.google.com/site/bioericscript/) and BREAKFAST (two different minimum distance cut-offs were used: 5 kb and 100 kb) (https://github.com/annalam/breakfast). STAR-Fusion showed higher sensitivity in detecting the fusions reported in previous TCGA studies. Therefore, we focused on the STAR-Fusion output and integrated EricScript and BREAKFAST output in one of the following filtering steps: 1) an exclusion list of genes was curated, including uncharacterized genes, immunoglobulin genes, mitochondrial genes, etc. Fusions involving these genes were filtered; 2) Fusions from the same gene or paralog genes (downloaded from https://github.com/STAR-Fusion/STAR-Fusion_benchmarking_data/tree/master/resources) were filtered; 3) Fusions reported in normal samples were filtered, including the ones from TCGA normal samples, GTEx tissues, and non-cancer cell study (Babiceanu et al., 2016); 4) For the fusions reported by only STAR-Fusion, a minimum value of FFPM > 0.1 (fusion fragments per million total reads) was required, as suggested by the authors; for the fusions reported by two or more callers, no minimum FFPM was required. 5) Finally, fusions with the same breakpoints in ≥ 10 samples across different cancer types were removed unless they were reported in previous TCGA studies.

#### Validation of Fusion Transcripts

For fusion events where low-pass whole genome sequencing data or WGS data were available from the ISB Cancer Genomics Cloud (https://isb-cgc.appspot.com/), we obtained high quality (-q 20) reads mapping to each partner gene and the 100kb region up and downstream using SAMtools. At least 3 discordant reads from WGS were required to determine if the fusion prediction was validated.

#### Gene Expression Analysis

We collected gene expression, copy number, and fusion annotations to test for associations between gene expression and fusion status. We used Tukey’s definition of outliers to determine if the expression level at a given gene was an outlier or not. An overexpression outlier means the sample’s expression level at a given gene was greater than (75th percentile) + 1.5*IQR, where IQR is the interquartile range. An underexpression outlier means the sample’s expression level at that gene was less than (25th percentile) −1.5*IQR. To test for a significant association between expression and fusion status, we calculated p values using both a t test and Fisher’s Exact Test. If either of those results passed stringent FDR multiple test correction, three or more fusions were reported, and if the median expression of the fusions was in the top or bottom decile of the data, we reported those genes for manual review.

#### Protein Kinase Fusion Analysis

We curated a list of kinase genes from previous publications and public databases (Table S5). Then we compared this list with UniProt/PFAM domain database (http://www.uniprot.org/database/DB-0073) to retain the ones with an annotated kinase domain. For the fusions involving kinase genes, we used AGFusion (https://github.com/murphycj/AGFusion) to check whether the annotated kinase domain was still present in the fusion transcript to separate them into fusions with an intact kinase domain versus those with a disrupted kinase domain. We compared the breakpoint positions in each fusion with the annotation file to check whether the breakpoint was in the 5′UTR, CDS, or 3′UTR region. Kinase genes are classified into eight groups: AGC, CAMK, CK1, CMGC, STE, TK, TKL, and others based on the PhosphoSite Database (Hornbeck et al., 2015). The percentage of kinase genes in each group across different cancer types was defined as the number of kinase genes with fusions in each group divided by their sum, denoted as *p_g_*. For each cancer type, the number of kinase genes in each group was first normalized by *p_g_*, denoted as *n_g_*. Then each number was divided by their sum *n_g_*/Σ*n_g_* to calculate a normalized percentage of kinase genes in each group.

#### Neoantigen Prediction

For each predicted fusion, we obtained translated protein sequences for novel transcripts from STAR-Fusion. The wild-type protein sequences are obtained from Ensembl Database. We constructed different epitope lengths (8–11-mer) from the translated protein sequence. Each sample’s HLA type comes from the TCGA Pan-Cancer Immune Group (Synapse ID: syn5974636). We predicted the binding affinity between epitopes and the major histocompatability complex (MHC) using NetMHC4 (Andreatta and Nielsen, 2016). Epitopes with binding affinity ≤ 500nM which are also not present in the wild-type transcript are reported as neoantigens. We required at least 5 splitting reads for supporting junctions to filter fusions with low expression.

#### Mutual Exclusivity Analysis

For TCGA tumor samples where both MC3 (Key Resources Table; Ellrott et al., 2018) mutation calls and gene fusion calls were available, we obtained the genetic alteration events, including fusion, inframe deletion, inframe insertion, missense mutation, nonsense mutation, nonstop mutation, splice site mutation, and translation start site mutation in 299 driver genes. We separated all the genomic alterations and events into “driver mutation,” “mutation,” and “fusion” categories, and compiled a genomic alteration profile for each sample. To test if the total number of mutations are significantly different among groups, we took samples without mutations in the following genes: *POLE, MLH1, MLH3, MGMT, MSH6, MSH3, MSH2, PMS1,* and *PMS2*, to exclude the confounding factor stemming from microsatellite instability. We then calculated p values by using Mann-Whitney U Test.

#### DEPO

DEPO is a curated list of druggable variants filtered such that each variant corresponds to one of several categories: single nucleotide polymorphisms or SNPs (missense, frameshift, and nonsense mutations), inframe insertions and deletions (indels), copy number variations (CNVs) or expression changes. Each variant/drug entry in DEPO was paired with several annotations of potential interest to oncologists. DEPO is available as a web portal (http://dinglab.wustl.edu/depo).

## Supplementary Material

1

2

3

4

5

6

7

## Highlights

- Highly recurrent fusions were found in prostate, bladder, breast, and lung cancers
- Expression increased in oncogene fusions but decreased in tumor suppressor genes
- Thyroid carcinoma showed significantly higher rates of kinase fusions
- Tumors with fusion events tend to have lower mutational burden
