# Supplementary material for: Driver Fusions and Their Implications in the Development and Treatment of Human Cancers
Source: Cell Rep. Author manuscript; Available in PMC 2018 Apr 25. (PMC5916809; doi:10.1016/j.celrep.2018.03.050)
Supplement: 1 [file NIHMS958978-supplement-1.pdf]

Cell Reports, Volume *23*

Supplemental Information

Driver Fusions and Their Implications in the Development and Treatment of Human Cancers

Qingsong Gao, Wen-Wei Liang, Steven M. Foltz, Gnanavel Mutharasu, Reyka G. Jayasinghe, Song Cao, Wen-Wei Liao, Sheila M. Reynolds, Matthew A. Wyczalkowski, Lijun Yao, Lihua Yu, Sam Q. Sun, The Fusion Analysis Working Group, The Cancer Genome Atlas Research Network, Ken Chen, Alexander J. Lazar, Ryan C. Fields, Michael C. Wendl, Brian A. Van Tine, Ravi Vij, Feng Chen, Matti Nykter, Ilya Shmulevich, and Li Ding

A

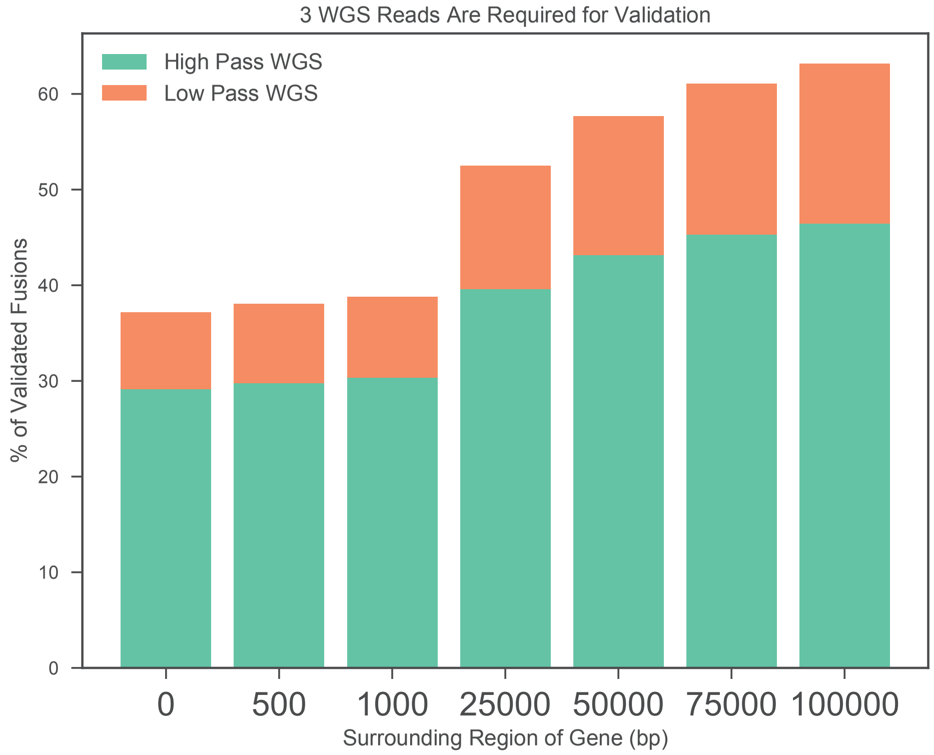

B

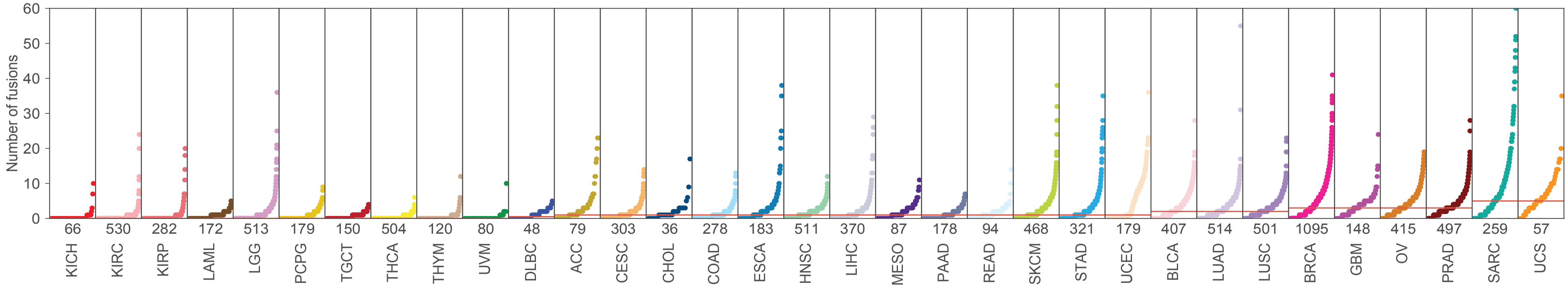

Figure S1 Fusion validation and landscape. Related to Figure 1. (A) Fusion events with available low-pass (low coverage) or high-pass whole genome sequencing (> 30x coverage). Fusions with WGS validation from high-pass are indicated in green and those from low-pass are in orange. (B) The dot plot illustrates the number of reported fusions per cancer type. Cancer types are indicated at the bottom, ordered by median number of fusions per sample.

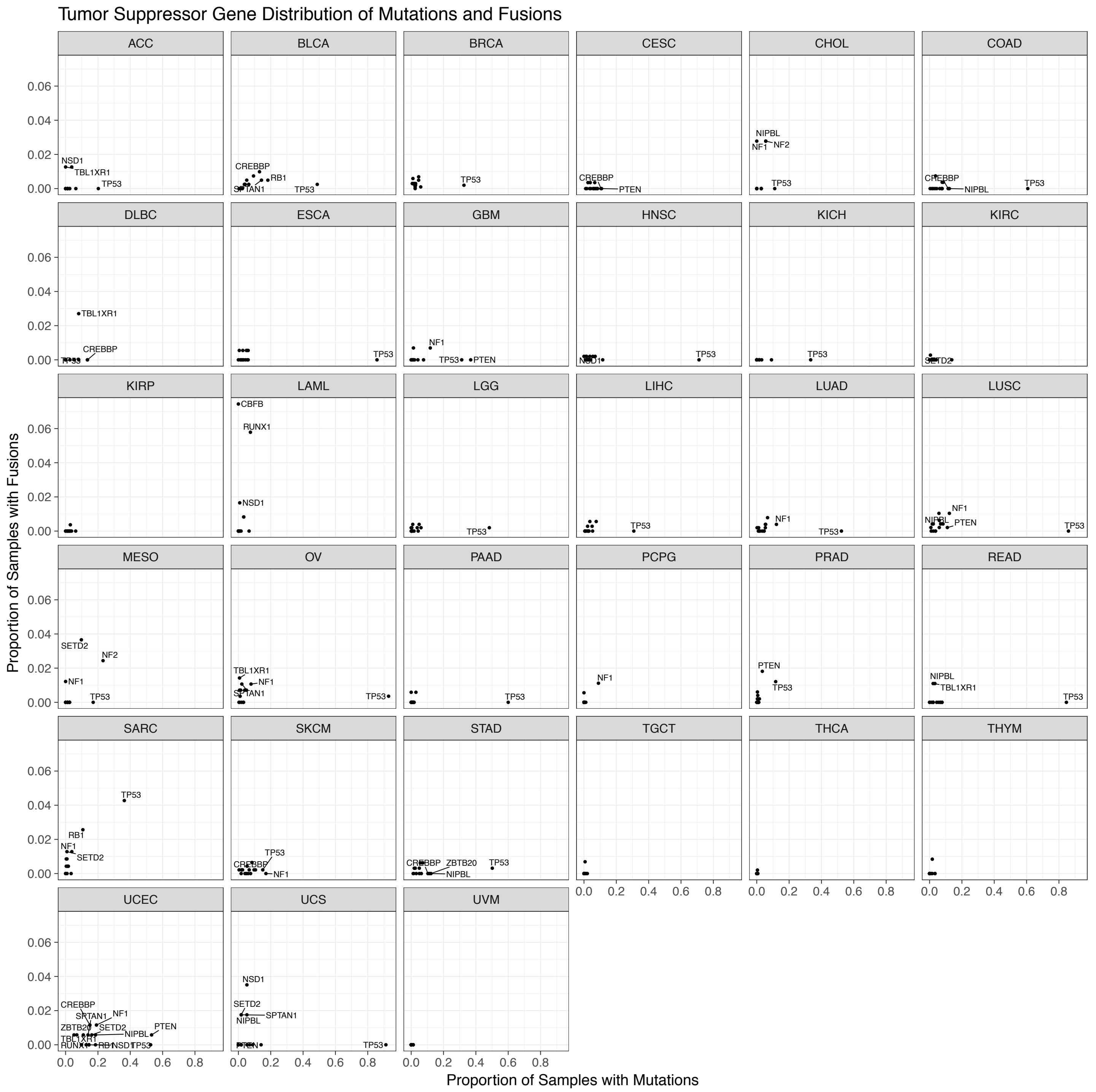

Figure S2 Tumor suppressor gene distribution of mutations and fusions. Related to Figure 2. Proportion of samples with mutations and fusions in tumor suppressor genes, separated by cancer type

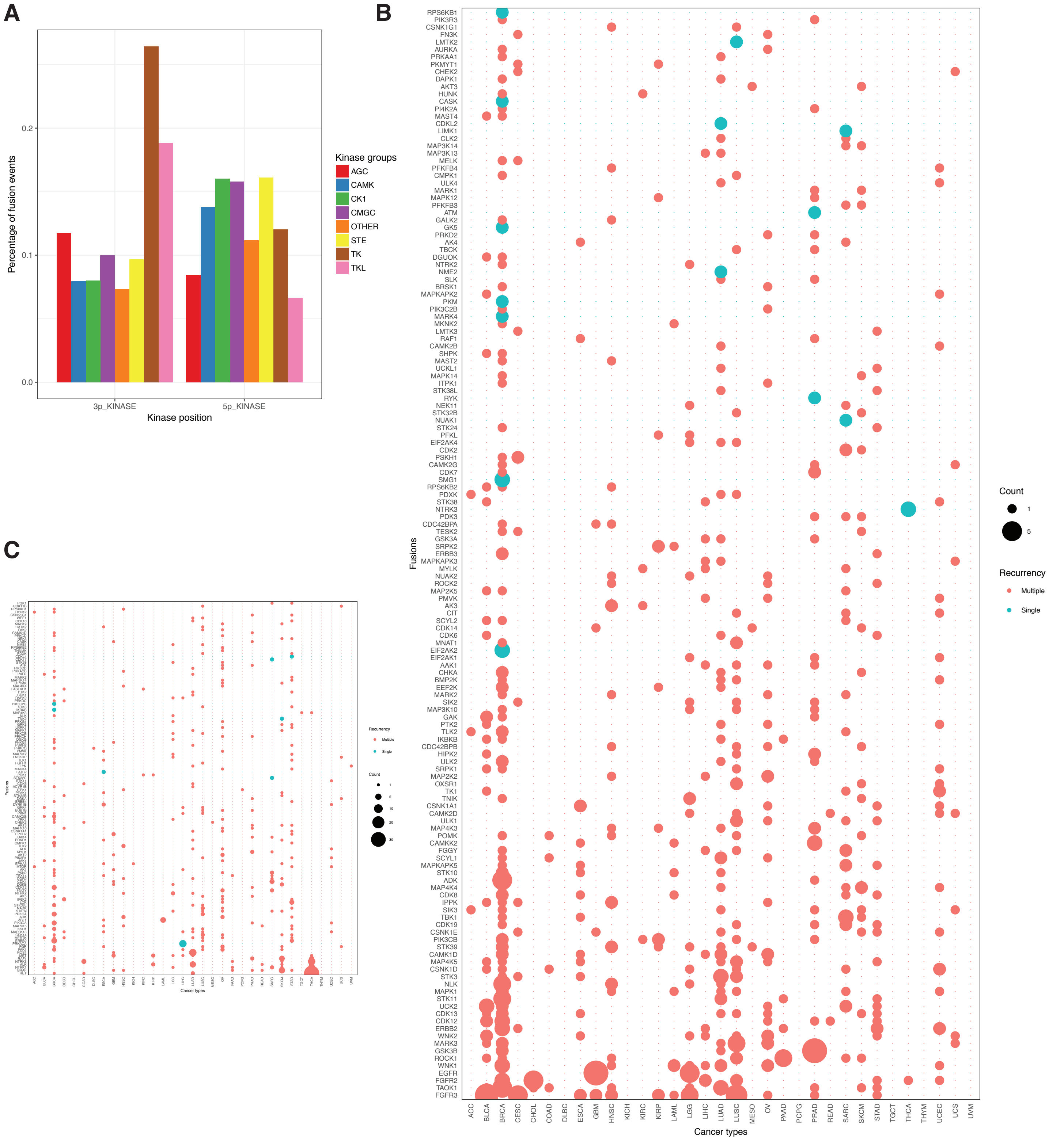

Figure S3 Protein kinase fusions. Related to Figure 3. (A) Normalized percentage of fusion events in different kinase groups for both 5'-kinase and 3'-kinase fusions. (B) The dot plot shows the numbers of samples for recurrent 5' kinase fusions. (C) The dot plot shows the numbers of samples for recurrent 3' kinase fusions.

A

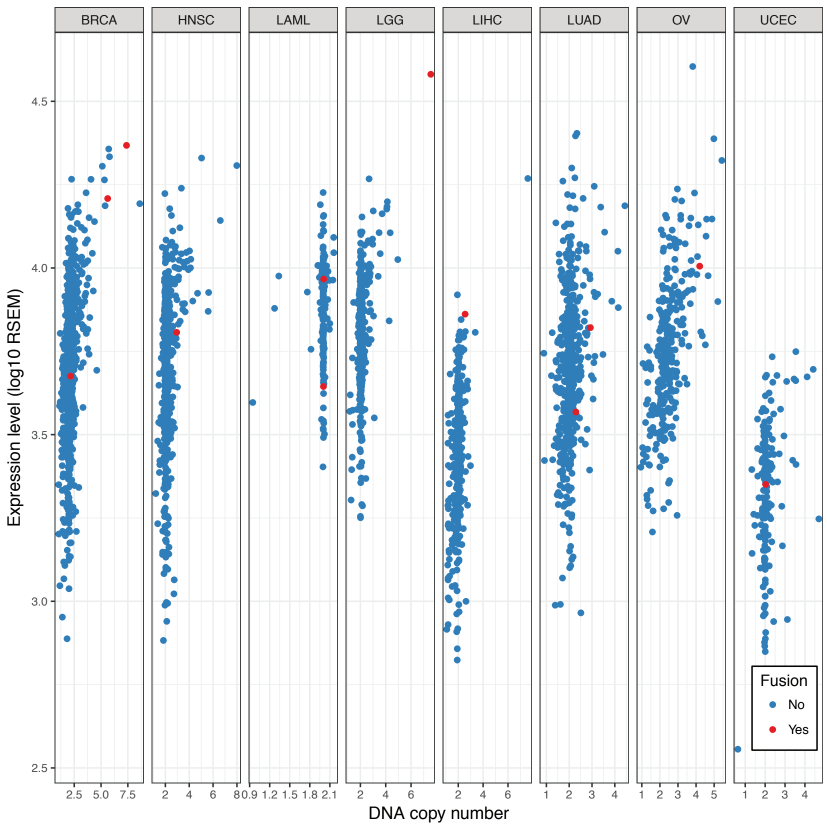

B

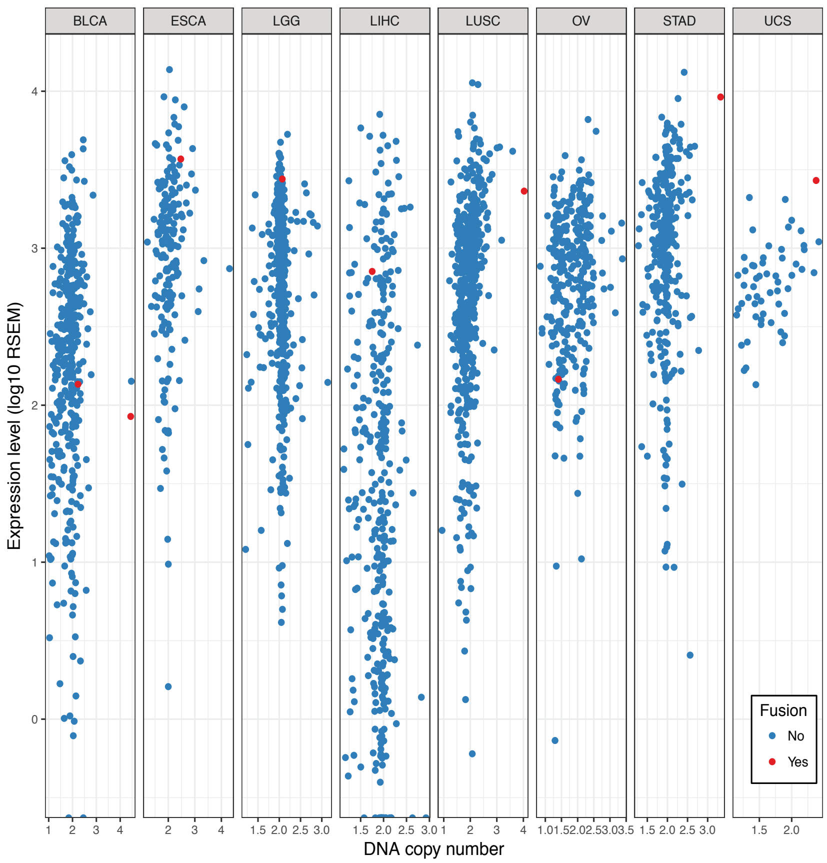

Figure S4 WNK Fusion. Related to Figure 3. (A) Scatter plots of WNK1 copy number versus mRNA expression across different cancer types. The samples with fusions are marked in red. (B) Scatter plots of WNK2 copy number versus mRNA expression across different cancer types. The samples with fusions are marked in red.

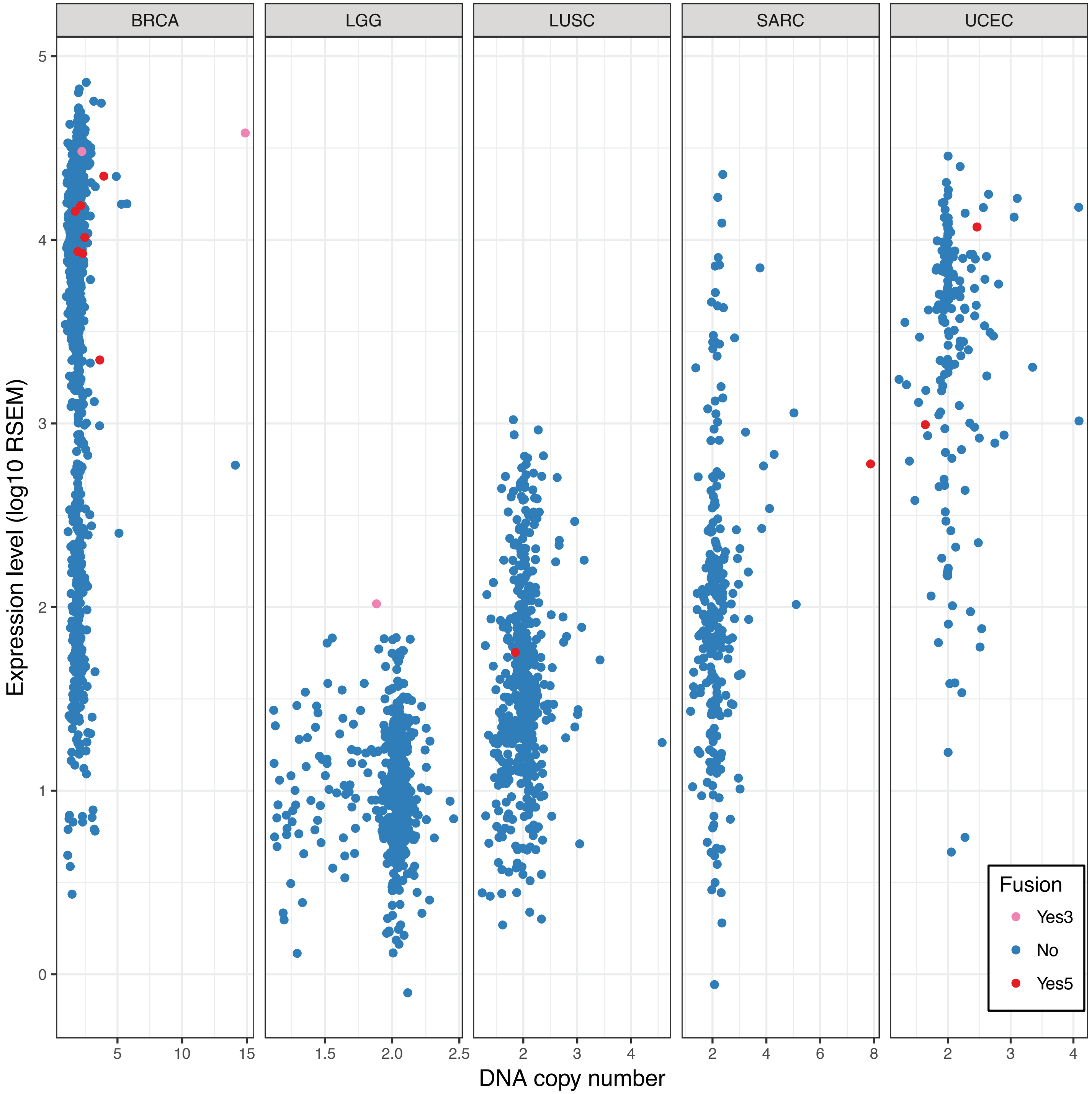

Figure S5 ESR1 Expression. Related to Figures 5 and 6. Expression level of ESR1 in samples across cancer types, with fusion status at 5' or 3' end.

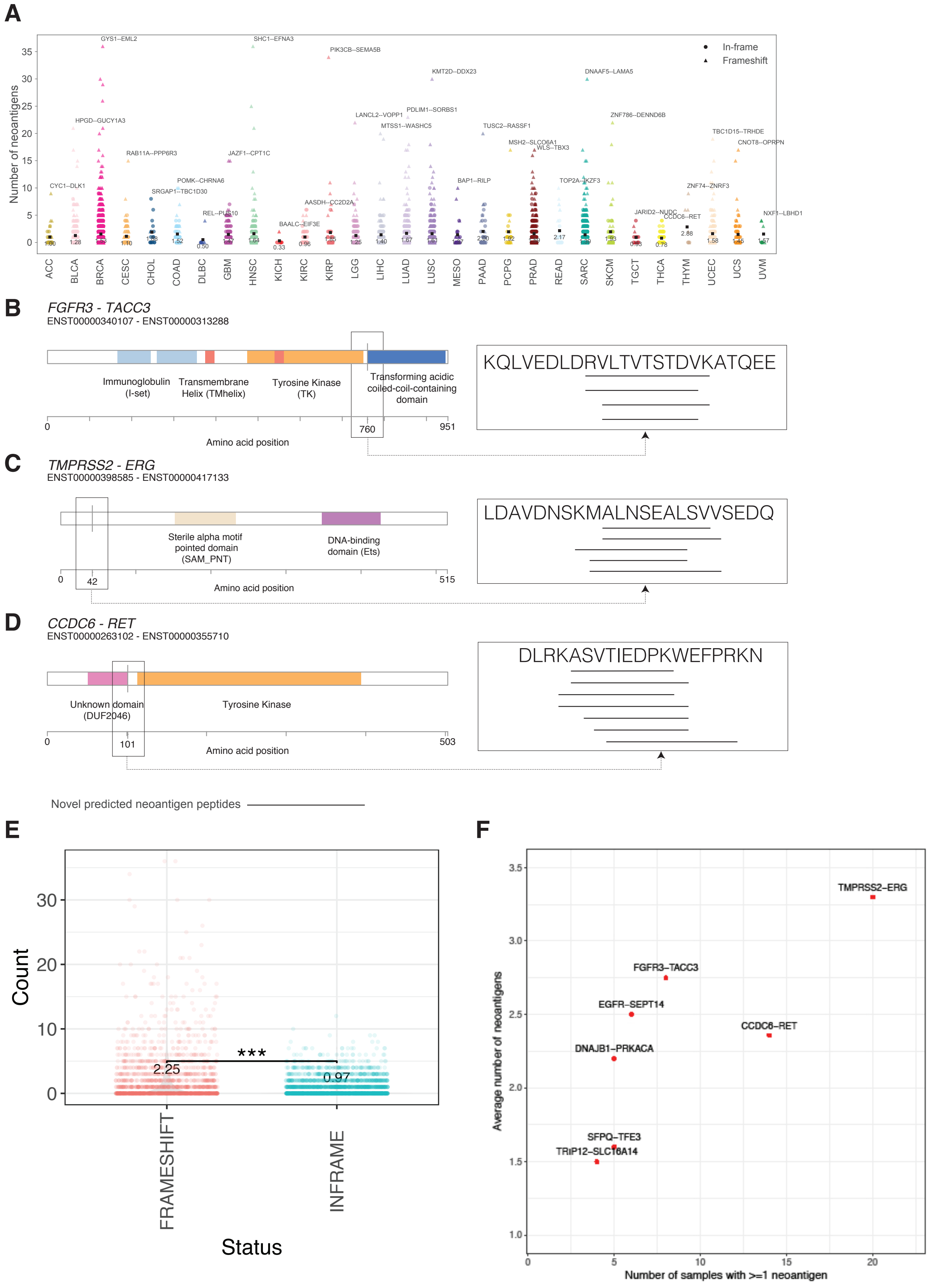

Figure S6 Fusion neoantigens. Related to Figure 6. (A) The distribution of the number of neoantigens for each fusion across samples and cancer types. (B), (C), and (D) are the unique neoantigen sequences detected across samples in three highly recurrent fusions FGFR3--TACC3, TMPRSS2--ERG, and CCDC6--RET. (E) Comparison of the number of predicted neoantigen between inframe and frameshift fusions. (F)The average number of neoantigens for these highly recurrent fusions found across cancer types (cut-off: the number of samples with neoantigen >= 4).
